# Posterior reversible encephalopathy syndrome (PRES) associated with SARS-CoV-2 infection in a patient under maintenance haemodialysis: a case report

**DOI:** 10.1186/s12882-023-03319-7

**Published:** 2023-09-29

**Authors:** Yuki Shimamoto, Hirohito Sasaki, Kenji Kasuno, Yuki Watanabe, Sayumi Sakashita, Sho Nishikawa, Kazuhisa Nishimori, Sayu Morita, Yudai Nishikawa, Mamiko Kobayashi, Sachiko Fukushima, Soichi Enomoto, Naoki Takahashi, Tadanori Hamano, Ippei Sakamaki, Hiromichi Iwasaki, Masayuki Iwano

**Affiliations:** 1 Department of Nephrology, Faculty of Medical Sciences, University of Fukui, 23-3 Matsuokashimoaizuki Eiheiji-cho Yoshida-gun, Fukui, Japan; 2 Division of Neurology, Second Department of Internal Medicine, Faculty of Medical Sciences, University of Fukui, Fukui, Japan; 3 Department of Infectious Diseases, Faculty of Medical Sciences, University of Fukui, Fukui, Japan; 4 Division of Infection Control and Prevention, University of Fukui Hospital, Fukui, Japan

**Keywords:** End-stage kidney disease, Haemodialysis, Intravascular endothelial cell injury, Posterior reversible encephalopathy syndrome, Status epilepticus

## Abstract

**Background:**

Endothelial dysfunction is common in patients undergoing chronic haemodialysis, and is a major cause of posterior reversible encephalopathy syndrome (PRES). Recently, Severe acute respiratory syndrome coronavirus 2 (SARS-CoV-2) has been shown to cause endothelial dysfunction by infecting vascular endothelial cells. Several cases of neurological complications in patients without kidney dysfunction, and only a few cases in patients with chronic kidney disease, have been reported in the literature. However, no previous report has yet described PRES associated with SARS-CoV-2 infection among patients undergoing maintenance dialysis.

**Case presentation:**

A 54-year-old woman undergoing maintenance haemodialysis was admitted to our hospital for status epilepticus. She had developed end-stage kidney disease (ESKD) secondary to diabetic nephropathy. Seven days prior to admission, she had developed fever and was diagnosed with COVID-19. Subsequently her blood pressure increased from 160/90 mmHg to 190/100 mmHg. On admission, she presented with severe hypertension (> 220/150 mmHg), unconsciousness, and epilepticus. CT tomography revealed no signs of brain haemorrhage. Cranio-spinal fluid (CSF) examination revealed no signs of encephalitis, and CSF polymerase chain reaction (PCR) for SARS-CoV-2 was negative. MRI findings revealed focal T2/FLAIR hyperintensity in the bilateral parietooccipital regions, leading to the diagnosis of PRES. Deep sedation and strict blood pressure control resulted in a rapid improvement of her symptoms, and she was discharged without sequelae.

**Conclusions:**

We report the first case of PRES associated with SARS-CoV-2 infection in a patient undergoing maintenance haemodialysis. Patients undergoing maintenance haemodialysis are at high risk of PRES because of several risk factors. SARS-CoV-2 infection causes direct invasion of endothelial cells by binding to angiotensin-converting enzyme 2 (ACE2), initiating cytokine release, and hypercoagulation, leading to vascular endothelial cell injury and increased vascular leakage. In the present case, SARS-CoV-2 infection possibly be associated with the development of PRES.

## Background

Severe acute respiratory syndrome coronavirus 2 (SARS-CoV-2) infection is associated with several neurological complications, including encephalitis, encephalopathies, acute neuropathies, myelitis, ischaemic stroke, and brain haemorrhage [1]. As patients infected with SARS-CoV-2 causes immune system dysregulation and hypercoagulable state, stroke is not uncommon, occurring in 2–6% of patients hospitalised with COVID-19 [1] Furthermore, intracranial haemorrhage occurs in approximately 20% of all COVID-19 cases [2].

Focal neurological deficits during SARS-CoV-2 infection indicate the most probable diagnosis of stroke; however, Posterior Reversible Encephalopathy Syndrome (PRES) should be a critical differential diagnosis. PRES is caused by the rapid development of subcortical vasogenic oedema, predominantly involving the parietooccipital region symmetrically [3]. Several risk factors for PRES have been suggested, including blood pressure fluctuations, autoimmune disorders, cytotoxic drugs, immunosuppressive drugs, eclampsia, and renal failure [4]. Among these factors, renal failure has been reported as the strongest predictor of PRES [5]. There have been several reported cases of chronic kidney disease (CKD) patients developing PRES associated with SARS-CoV-2 infection [6, 7]. Nevertheless, there have been no reports of haemodialysis patients developing PRES associated with SARS-CoV-2 infection. Therefore, in this article, we report the first case of PRES associated with SARS-CoV-2 infection in a haemodialysis patient.

## Case presentation

### Clinical presentation

A 54-year-old Japanese woman with end-stage kidney disease (ESKD) caused by diabetic kidney disease was transferred to our hospital for generalised seizures. She had started undergoing haemodialysis (HD) six months prior to admission to the hospital.

Seven days prior to admission, she developed fever and was diagnosed with COVID-19. She was prescribed 1,600 mg/day of molnupiravir, but she could not take them because of nausea. Her usual home blood pressure was around 160/90 mmHg, but was elevated to approximately 190/100 mmHg following diagnosis with COVID-19 by nasopharyngeal swab PCR (Ct value: 15.3). She became afebrile after 2 days.

On arrival at the hospital, her GCS score was 8 (E3 V2 M3) and blood pressure exceeded 220/150 mmHg. Because of relapsing generalised seizures, we administered 10 mg diazepam a total of six times. Physical examination revealed a temperature of 37.0 °C, heart rate of 114 beats/min, blood pressure of 225/132 mmHg, and oxygen saturation of 99% in room air. A right-sided conjugate deviation was observed during the seizures. Cardiovascular, respiratory, and abdominal examinations revealed no complications. Her dry weight (DW) was calculated as 52.0 kg, but her actual body weight was 50.5 kg on admission.

Laboratory data revealed white blood cells 8,700 /µL, hemoglobin 12.9 mg/dL, platelets 84,000 /µL, sodium 141 mEq/L, potassium 5.1 mEq/L, chloride 104 mEq/L, calcium 8.6 mg/dL, phosphorus 5.4 mg/dL, serum creatinine 9.03 mg/dL, blood urine nitrogen 37 mg/dL, blood glucose 246 mg/dL, C-reactive protein 0.01 mg/dL, Neutrophil-to-lymphocyte ratio 15.9, and AST/ALT ratio 2.1. The patient’s creatinine kinase level was elevated to 2817 U/L. Arterial blood gas analysis revealed pH 7.258, pO2 91.9 mmHg, pCO2 49.5 mmHg, HCO3 21.4 mmol/L, and lactate 13 mg/dL. Endocrinology testing showed TSH 0.842 µIU/mL, FT4 1.03 ng/dL, and FT3 < 1.50 pg/mL. Computed tomography scan of chest and brain revealed slight bilateral ground-glass opacities in the peripheral area of the lung, while brain haemorrhage was not detected. Cerebrospinal fluid (CSF) examination revealed a normal cell count, total protein 25 mg/dL, glucose 120 mg/dL, IgG index 0.52, and adenosine deaminase (ADA) < 1.0 U/L, showing no evidence of meningitis or encephalitis. The CSF culture was negative. SARS-Cov-2 PCR of nasopharyngeal swabs was positive, while PCR of samples in the CSF was negative.

### Imaging

**Fig. 1:**
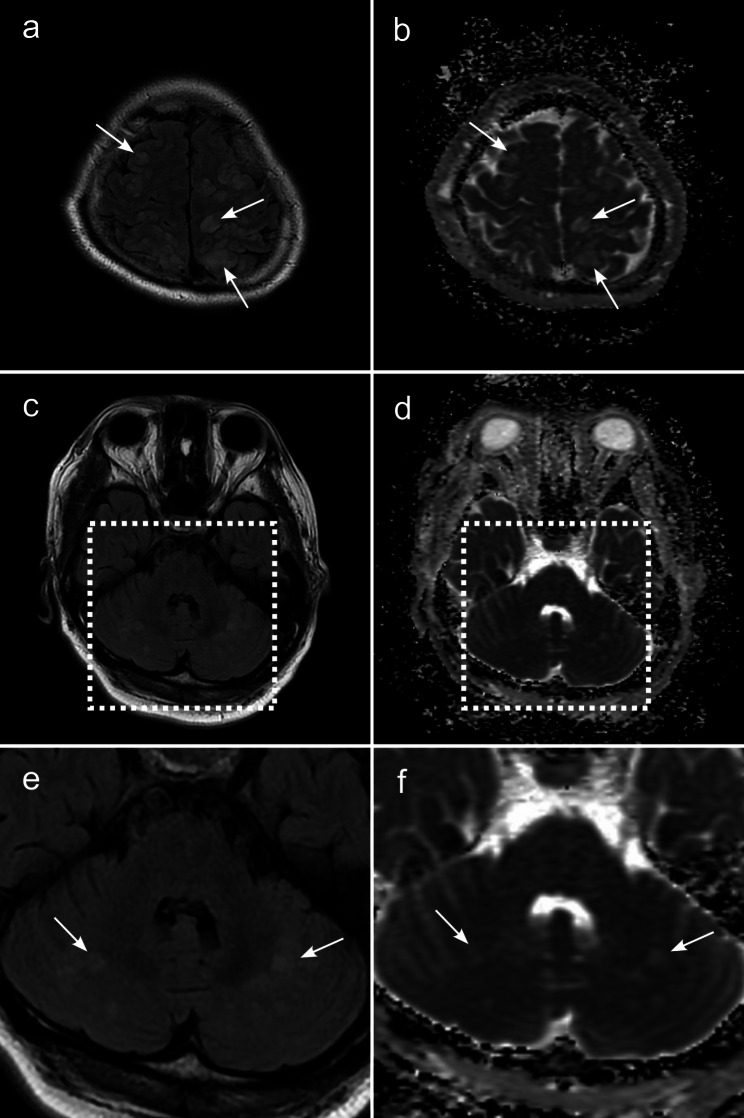
MRI findings on admission Bilateral multifocal hyperintensities in the cortical and subcortical areas of the parietal lobe **(a)** and cerebellum **(c)** could be seen on FLAIR. ADC mapping hyperintensities are present in the same region as FLAIR **(b, d)**, suggesting that these changes were vasogenic oedema. High magnification of cerebellar abnormalities in FLAIR and ADC mapping **(e, f)**

**Fig. 2:**
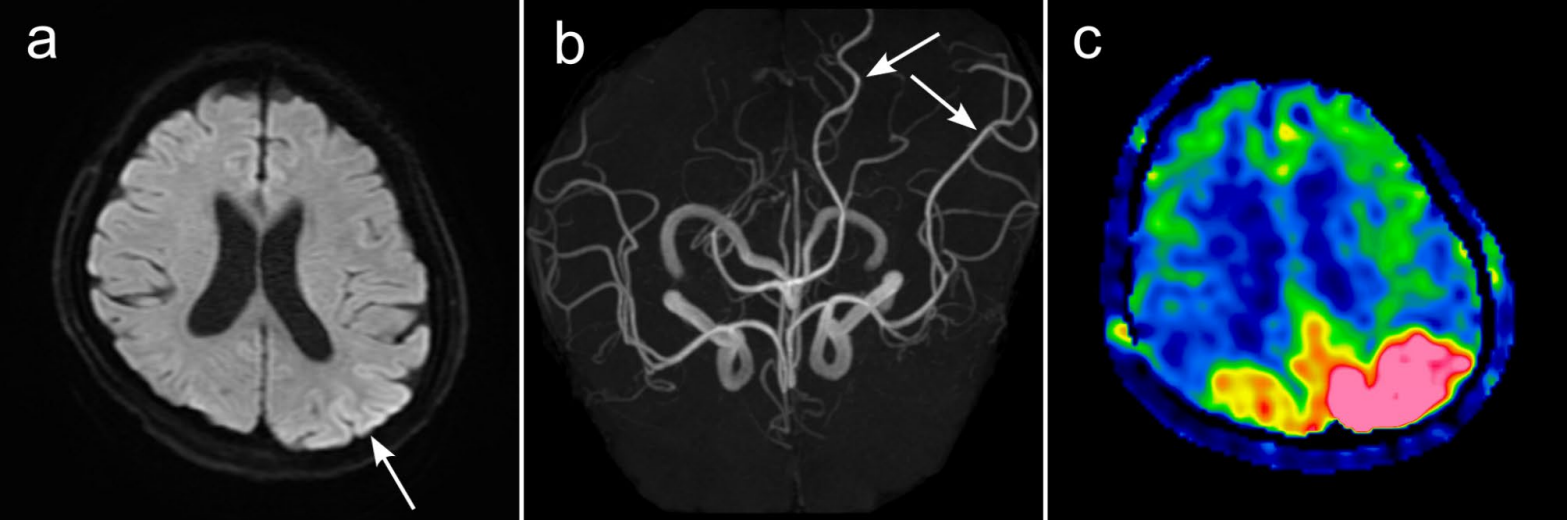
MRI and MRA findings on admission DWI hyperintensities are present in the left occipital lobe **(a)**, and the left MCA and left PCA are dilated **(b)**. ASL/CBF revealed increased blood flow in the same region **(c)**, suggesting that the left parietal lobe was the focus of epilepsy

MRI revealed multiple focal regions with T2 and fluid-attenuated inversion recovery (FLAIR) hyperintensities bilaterally in the cortical and subcortical areas of the parieto-occipital lobe and bilateral cerebellum (Fig. 1a, c and e). Apparent diffusion coefficient (ADC) mapping hyperintensities with the same regions as FLAIR were present, indicating that these changes were caused by increased vascular permeability, subsequently leading to angioedema (Fig. 1b, d and f). Diffusion-weighted imaging (DWI) revealed hyperintensity in the left occipital lobe (Fig. 2a) and dilatation of the left posterior cerebral artery (PCA) and left middle cerebral artery (MCA) on magnetic resonance angiography (MRA) (Fig. 2b). Focal hyperintensity was observed in the left parietal region, with arterial spin labelling/cerebral blood flow (ASL/CBF) (Fig. 2c). DWI, MRA, and ASL/CBF findings indicated that the left parietal lobe was the focus of epilepticus. These findings suggested a diagnosis of PRES and post-epileptic changes. Intracranial haemorrhage was not detected on CT or T2 star-weighted images.

### Clinical course

She was initially intubated with deep sedation for status epilepticus. After admission to the intensive care unit, blood pressure was controlled with continuous intravenous nicardipine. Remdesivir was initiated at an initial dose of 200 mg, then followed by 100 mg/day for four consecutive days. For suspected encephalitis, a methylprednisolone pulse of 1,000 mg/day for three consecutive days and 500 mg/day levetiracetam were administered. Seizure relapsed after tapering off the propofol on day 3. Dose of levetiracetum was increased to 1000 mg/day on day4, with addition of lacosamide 100 mg/day on day 5. The seizures control was achieved with the two antiepileptics and she was extubated on day 8.

**Fig. 3:**
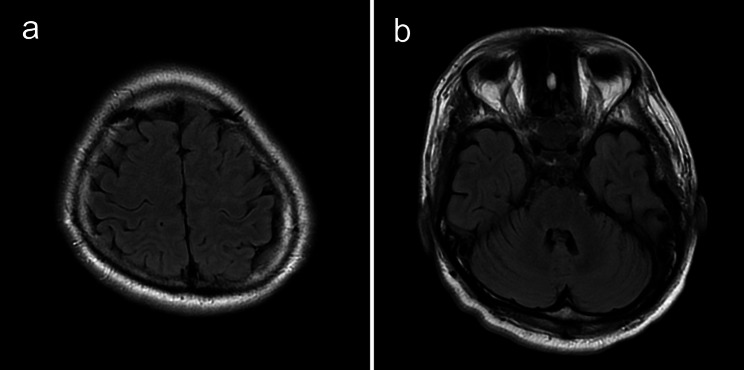
Resolution of abnormal findings on MRI on day 15 Multifocal hyperintensities of the parietal lobe **(a)** and cerebellum **(b)** are completely resolved

Follow-up MRI on day 15 revealed that the bilateral multifocal T2/FLAIR hyperintensities and ASL hyperintensities in the left hemisphere had diminished (Fig. 3). Reversible changes on MRI confirmed the diagnosis of PRES and post-convulsive changes. Electroencephalography performed on day 15 of admission did not reveal epileptic discharge.

Although encephalitis was unlikely the cause of the seizure, levetiracetam (500 mg/day) and lacosamide (100 mg/day) was continued due to status epilepticus on admission. Since seizure recurrence was not observed, the patient was discharged 7 weeks later with no sequelae.

## Discussion

We report as rare presentation of a haemodialysis patient who developed PRES associated with a COVID-19 infection. The diagnosis was confirmed based on the clinical course, reversible MRI findings, and negative CSF PCR for SARS-CoV-2.

As there are no established diagnostic criteria, PRES is usually diagnosed based on clinical and radiological findings after excluding other possible causes [8]. On admission, SARS-CoV-2 related encephalitis was suspected, as some cases of COVID-19 encephalopathy, encephalitis, or meningitis confirmed by CSF PCR have been reported [9, 10]. Further analysis of CSF PCR was done which was negative in this case. No other causative microorganism was detected in the CSF culture. Encephalitis of other causes was also excluded as none of the patient’s medical history indicated an association with other causes. MRI changes in the present case were observed not only in occipital and parietal lobes, but also in cerebellum. These multifocal lesions were similar to previous report of PRES in patients with ESKD [11]. Seizure and MRI changes were resolved by immediate blood pressure control, strongly supporting the diagnosis of PRES. It has been implied that PRES associated with SARS-CoV-2 infection presents a mildly higher risk of intracranial haemorrhage than PRES without SARS-CoV-2 infection [12], but the present case did not show any signs of haemorrhage.

Although the underlying mechanism has not been fully elucidated, renal failure is the strongest predictor of PRES [5]. Patients with renal failure experience endothelial dysfunction due to uremia [13]. Endothelial cells contribute to the maintenance of blood pressure by synthesising humoral factors such as nitric oxide (NO), smooth muscle vasodilator and endothelin-1 [14]. During haemodialysis, patients with hypertension show an increase in endothelin-1 levels, while NO remains disproportionately low [15], leading to vasoconstriction. Dysfunction of the endothelial cell response to haemodialysis is known among patients who receive beta-blockers, which suppress endothelin-1 release [16]. These factors cause hypertension in haemodialysis patients, which is a strong risk factor for PRES. Furthermore, endothelial cell dysfunction causes dysregulation of cerebral autoregulation, and may lead to extravasation of fluid at a much lower blood pressure, leading to vasogenic oedema [17]. There have been several cases of PRES in patients with renal failure who presented with normotensive blood pressure [7, 18]. Therefore, endothelial cell dysfunction in haemodialysis patients is a strong risk factor for PRES. Another well-known cause of hypertension in haemodialysis patients is erythropoietin-stimulating agents (ESA). ESA administration corrects anaemia, which leads to hypoxic vasodilation and increased blood viscosity. These changes result in increased total peripheral resistance and subsequent hypertension [19]. Endocrinological changes, such as enhanced noradrenergic and angiotensin II sensitivity and endothelin-1 concentration, have also been suggested to play a role [20]. The present case showed a relatively high haemoglobin level on admission; however, her body weight was 1.5 kg lower than her dry weight. This implies that she was experiencing volume deletion and Hb levels were elevated by haemoconcentration. Because her blood pressure had increased following diagnosis with COVID-19, we surmised that the main cause of hypertension was not ESA.

Acute inflammatory responses to several infectious pathogens are known to cause endothelial cell injury and immuno-thrombosis [21]. In addition to the common mechanism of infectious intravascular endothelial cell injury, SARS-CoV-2 infection has a unique pathogenesis. First, SARS-CoV-2 strongly binds to and downregulates ACE2 receptors [22]. ACE2 is innately expressed on the surface of intravascular endothelial cells, where it removes a single amino acid from the octapeptide angiotensin II and converts it into angiotensin1-7. Since angiotensin1-7 plays a key role in vascular smooth muscle cell dilation and suppressive effects on coagulation, downregulation of ACE2 is associated with hypertension and hypercoagulation. ACE2 is also known to mediate viral entry into cells [23]. SARS-CoV-2 has been detected in several different organs of deceased patients [24]. Research has shown that ACE2 downregulation may play a key role in the pathogenesis of SARS-CoV-2 infections. SARS-CoV-2 infection causes intravascular endothelial cell lysis and death leading to vascular leakage [23]. It has also been clinically demonstrated that blood pressure is elevated in patients with SARS-CoV-2 infection compared to non-infected patients [25]. In our case, diabetes and hypertension, which are both known risk factors for ACE2 deficiency and SARS-CoV-2 infection, led to further depletion of ACE2 function and hypertension.

Second, SARS-CoV-2 infection increases vascular permeability. SARS-CoV-2 infects vascular endothelial cells and alters gene expression, leading to vascular endothelial cell dysfunction [26]. Downregulation of ACE2 activity indirectly activates the kallikrein-bradykinin pathway, leading to increased vascular permeability [27]. Neutrophils and monocytes are activated and recruited to generate reactive oxygen species (ROS). Furthermore, immune cells release inflammatory cytokines and vasoactive agents which trigger loosening of the tight junction of intravascular endothelial cells [23]. In addition, cytokines such as Tumor necrosis factor (TNF) and IL-1-beta activate glucuronidases that degrade the glycocalyx, while these cytokines increase hyaluronic acid synthase 2, which increases hyaluronic acid deposition in the extracellular matrix and increased vascular permeability [23]. As the pathogenesis of PRES involves increased vascular permeability and brain oedema, it is strongly indicated that SARS-CoV-2 infection was closely associated with the development of PRES in the present case.

We present a rare case of PRES associated with COVID-19 that developed in a haemodialysis patient. In addition to renal failure and haemodialysis, SARS-CoV-2 infection possibly associated with hypertension and increased blood vessel permeability in such patients.

## Data Availability

Y.S. and corresponding author have full access to the data in this report. Anonymized data can be provided upon reasonable request (kasuno@u-fukui.ac.jp).

## Abbreviations

PRES: posterior reversible encephalopathy syndrome
SARS-CoV-2: severe acute respiratory syndrome coronavirus 2
ESKD: end-stage kidney disease
CSF: cerebrospinal fluid
PCR: polymerase chain reaction
DW: dry weight
MRI: magnetic resonance imaging
FLAIR: fluid attenuated inversion recovery
CKD: chronic kidney disease
HD: haemodialysis
Ct: Threshold cycle
GCS: Glasgow coma scale
ASL: arterial spin labelling
PCA: posterior cerebral artery
NO: nitric oxide
ESA: erythropoietin-stimulating factor
ACE2: angiotensin-converting enzyme 2
ROS: reactive oxygen species
IL-1: interleukin-1
TNF: tumour necrosis factor
